# Distinct Interactions between Fronto-Parietal and Default Mode Networks in Impaired Consciousness

**DOI:** 10.1038/srep38866

**Published:** 2016-12-13

**Authors:** Jinyi Long, Qiuyou Xie, Qing Ma, M. A. Urbin, Liqing Liu, Ling Weng, Xiaoqi Huang, Ronghao Yu, Yuanqing Li, Ruiwang Huang

**Affiliations:** 1 Collage of Information Science and Technology, Jinan University, Guangzhou 510632, China; 2 Center for Brain Computer Interfaces and Brain Information Processing, South China University of Technology, Guangzhou 510640, China; 3 Coma Research Group, Centre for Hyperbaric Oxygen and Neurorehabilitation, Guangzhou General Hospital of Guangzhou Military Command, Guangzhou 510010, China; 4 Program in Physical Therapy, Washington University School of Medicine, St. Louis, MO 63108, USA; 5 Brain Imaging Center, Center for Studies of Psychological Application, School of Psychology, South China Normal University, Guangzhou 50016, China; 6 Huaxi MR Research Center, Department of Radiology, West China Hospital of Sichuan University, Chengdu 610041, China

## Abstract

Existing evidence suggests that the default-mode network (DMN) and fronto-pariatal network (FPN) play an important role in altered states of consciousness. However, the brain mechanisms underlying impaired consciousness and the specific network interactions involved are not well understood. We studied the topological properties of brain functional networks using resting-state functional MRI data acquired from 18 patients (11 vegetative state/unresponsive wakefulness syndrome, VS/UWS, and 7 minimally conscious state, MCS) and compared these properties with those of healthy controls. We identified that the topological properties in DMN and FPN are anti-correlated which comes, in part, from the contribution of interactions between dorsolateral prefrontal cortex of the FPN and precuneus of the DMN. Notably, altered nodal connectivity strength was distance-dependent, with most disruptions appearing in long-distance connections within the FPN but in short-distance connections within the DMN. A multivariate pattern-classification analysis revealed that combination of topological patterns between the FPN and DMN could predict conscious state more effectively than connectivity within either network. Taken together, our results imply distinct interactions between the FPN and DMN, which may mediate conscious state.

Previous neuroimaging studies have shown that a core set of brain regions within the fronto-parietal network (FPN) and the default mode network (DMN) is centrally involved in conscious processes^1^,^2^. Functional connectivity analyses have demonstrated internetwork coupling between networks is necessary to support conscious cognitive processes^3^,^4^. How functional networks operate and interact with each other in pathological conditions characterized by impaired consciousness is not well understood.

Unlike traditional task-evoked paradigms, functional magnetic resonance imaging in the resting state (R-fMRI) is particularly suitable for studying the brain functions of individuals with disorders of consciousness (DOC). Multiple R-fMRI studies demonstrated that nodal topology is disrupted in impaired consciousness^5^,^6^,^7^. Specifically, whole-brain network efficiency, measured by the shortest path length and modularity, is altered in different brain regions depending on conscious state^5^,^6^. For instance, Crone *et al*. found decreased local efficiency in several brain regions (e.g., medial parietal, frontal, thalamic) when comparing minimally conscious and unconscious patients^6^. Achard *et al*. examined functional networks in comatose patients shortly after brain injury and demonstrated extensive reorganization of central hubs, including precuneus of the DMN^5^. These studies, however, found no difference in global network topology between comatose patients and controls^5^ or between patients in a minimally conscious state (MCS) and vegetative/unresponsive wakeful state (VS/UWS)^6^. Another study investigated internetwork connectivity and found cortico-cortical anti-correlation between the FPN and DMN in a VS/UWS patient but not in a brain dead patient^3^, suggesting that the anti-correlation between the FPN and DMN may be preserved at the network level in impaired consciousness. Whether the interaction between these networks mediates consciousness and the specific network-level architecture involved remains unknown.

The purpose of this study was to more closely examine differences in connectivity patterns across conscious states and identify specific interactions between the FPN and DMN. This was accomplished by quantifying network level topological properties using R-fMRI in a cohort of DOC patients, including those in a VS and MCS. We hypothesized the differences would exist between conscious states in the topological properties of the FPN and DMN. We also hypothesized that the functional connectivity patterns between these two networks could discriminate conscious state.

## Materials and Methods

### Subjects

Twenty-four DOC patients (13 VS/UWS and 11 MCS) and 13 control subjects were recruited for this study. Six DOC patients (2 VS/UWS and 4 MCS) and 2 control subjects had to be excluded from the fMRI analysis due to excessive head movement (see the criteria described below)^8^. Controls were matched to DOC patients on age (*p* = 0.53) and gender (*p* = 0.28) (Table 1). In patients, hypoxic ischemic encephalopathy was the primary cause of brain injury (9 VS/UWS and 6 MCS). The clinical severity of each patient was assessed using the Coma Recovery Scale-Revised (CRS-R)^9^. This assessment was administered the day of scanning, as well as a week before and two weeks after scanning. Each patient was not sedated during image acquisition and no other medications were administered four hours before scanning. Structural MRI scans were acquired for clinical diagnosis and to identify and exclude subjects with larger hematoncus and malacostic foci, moderate to severe hydrocephalus, or severe cerebral atrophy. Exclusion criteria for DOC patients were as follows: (1) history of psychological disorders, (2) previous alcohol or drug abuse, (3) brain tumor, (4) obvious brain shrinkage or morphological changes, or (5) frequent spontaneous movements. The demographic and clinical characteristics for all subjects are listed in Table 1. This study was approved by the local Research Ethics Committee of Guangzhou General Hospital of Guangzhou Military Command, and all protocols were carried out in accordance with the Declaration of Helsinki. Written informed consent was obtained directly from each healthy subject and from the legal surrogate of each patient.

### Data Acquisition and Preprocessing

Brain functional and high-resolution structural images were acquired via a GE 3-T MR scanner housed in the Guangzhou General Hospital of Guangzhou Military Command. All subjects were instructed to lie motionless in the scanner with their eyes open. During data acquisition, head movements were minimized using customized cushions. Eye opening could not be fully controlled during the entire scanning procedure involving patients because of their cognitive and physical impairments^4^. If a patient was unable to remain motionless or closed their eyes, then the procedure was stopped and resumed at a later time. R-fMRI data were obtained using a single-shot gradient-echo EPI sequence (repetition time (TR) = 2000 ms, echo time (TE) = 50 ms, flip angle = 90°, matrix size = 64 × 64, voxel size = 3.6 × 3.6 × 3.6 mm^3^, 36 axial interleaved slices covering the whole brain, and 240 volumes acquired in 8 min). High-resolution brain structural images were acquired using a T1-weighted 3D MP-RAGE sequence (inversion time = 1100 ms, TR = 8.86 ms, TE = 3.52 ms, flip angle = 90°, matrix size = 256 × 256, voxel size = 1 × 1 × 1 mm^3^, and 176 sagittal slices) for co-registration with the functional images. R-fMRI data were preprocessed using SPM8 (http://www.fil.ion.ucl.ac.uk/spm/). The first five volumes for each subject were excluded before performing slice timing, head-motion correction, co-registration with the T1-weighted structural images, and normalization to the MNI template without spatial smoothing. Data from subjects with >3 mm/° of head movement/rotation during scan acquisition were excluded. Preprocessed images were de-trended to abandon linear trends and then temporally filtered with a Chebyshev band-pass filter (0.01–0.08 Hz).

### Functional Connectivity Estimation

To construct whole-brain functional networks for each subject, we used a brain partition scheme, Power-264 template that included 264 putative functional regions proposed by Power *et al*. according to a fMRI meta-analysis, to define nodes^10^. For a given brain region, the nodal mean time series was calculated by averaging all voxels in the region. Each nodal mean time series was corrected by regressing out head motion. Recent studies have regressed out global signal in functional connectivity analysis^11^,^12^,^13^, but global signal regression could mathematically create artefactual negative correlations. However, this technique can be used to effectively reduce motion-related artifacts that cannot be removed by a variety of motion-based regressors^12^ and is considered to be a processing maneuver that facilitates the evaluation of underlying neurophysiological relationships^11^, making the interpretation of the results with global signal regression more reliable. Each nodal mean time series, therefore, was corrected by regressing out the global signals. To further reduce spurious variance unlikely to reflect neuronal activity, other confounding factors were also regressed out, including the signals of cerebrospinal fluid (CSF) and white matter, as well as the first-order derivative terms of the global, white matter, and CSF signals^11^,^12^,^14^. Using the residual time series, we calculated Pearson’s correlation coefficient (subsequently Fisher’s *z* transformed) between each pair of regions to obtain inter-nodal connectivity, and to construct a brain network for each subject *g* = (*V, E*), where *V* corresponds to the set of the 264 nodes and *E* to the set of edges.

### Whole-Brain Network Partition and Network Analysis

**Figure 1:**
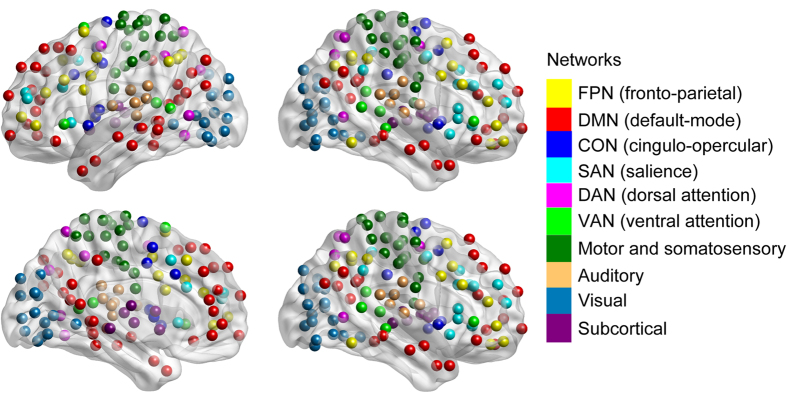
Illustration of 264 regions belonging to 10 whole-brain functional networks.

The brain partition scheme was obtained from a fMRI meta-analysis adopted in previous studies^10^,^15^. Similar to these previous studies, 264 regions of Power-264 template were partitioned into ten functional modules representing major networks (Fig. 1). The threshold of connection density (i.e., percentage of connections/edges) is an important parameter which affects characteristics of network topology while constructing brain networks for each subject^6^,^7^. A range of thresholds for connection density, therefore, were selected according to the following two criteria: 1) unconnected nodes <10% to guarantee that the resulting networks could be estimated^6^,^16^, and 2) small-worldness >1.5 to ensure that all thresholded networks had small-world properties and had as few spurious edges as possible^6^. As a result, the threshold range of connection density over which all whole-brain networks met the constraints was in the range of 2.5–32.5% in connections/edges with a step size of 2%. For a given threshold of this range, the absolute values of correlation coefficients were first sorted from high to low values. Edge weight was then set as the absolute value of correlation if this value was at the portion of the chosen threshold; otherwise, edge weight was set to zero^17^.

To characterize the ten networks and their specific interactions, topological metrics were computed including connectivity strength, betweenness, and degree, which are suggested to be important measures for examining the interactions between network elements (e.g., nodes) in network topology analysis^18^,^19^. This was done using the BCT toolbox (https://sites.google.com/site/bctnet/). For each of these topological metrics, its average value across all nodes within any of the ten networks was calculated and defined as the network level topological metric. Connectivity strength for the *i*th node is defined as follows:


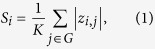


where *K* is the number of nodes in the whole-brain network *G*, and *z*_*i,j*_ is the *z*-score of the temporal correlation coefficient between the time courses of nodes *i* and *j*. For the *j*th (*j* = 1, 2, …, 10) network (*G*_*j*_), its connectivity strength can be calculated as:


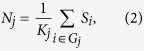


where *K*_*j*_ is the number of nodes in the *j*th network *G*_*j*_.

The global metric of characteristic path length and the local metric of clustering coefficient were calculated to examine the small-world property of a whole-brain network with a selected threshold. In accordance with previous work, these two metrics were first normalized to their corresponding values obtained by averaging 100 random networks with matched size and degree distribution of a brain network^5^,^6^,^20^. Next, the ratio of normalized clustering coefficient and normalized characteristic path length was calculated as the small-worldness of a whole-brain network. Compared to random networks, small-world networks were those with a higher degree of clustering and with an equal characteristic path length. Thus, the small-worldness of a network for a chosen threshold should be larger than 1. More detail about using this ratio to examine small-world characteristics has been reported previously^6^,^20^.

Other metrics (i.e., connectivity strength, betweenness, and degree) were also normalized to their corresponding metrics estimated in the randomized networks to characterize the topology of the ten networks and their specific interactions. For each of the above mentioned network metrics, the area-under-the-curve (AUC) method was used to summarize the results across the whole range of the selected connection density^5^,^6^.

### Distance-Related Connectivity Strength Analysis

Connectivity strength is suggested to be strongly associated with the spatial distance of a given inter-nodal connection^21^,^22^. Thus, it is important to characterize whether network interactions related to connectivity strength are distance dependent. This was addressed by measuring connectivity strength of each node within a given network to one of the other nodes within the whole-brain network and calculating the Euclidian distance between these two nodes’ centers. Given that there is currently no definitive threshold distance at which a given functional connection can be classified as short- or long-range, a 75 mm threshold used in previous work was adopted^23^,^24^. The distance was subdivided into 18 intervals of 10 mm ranging from 0 to 180 mm, which was the longest spatial distance between nodes. Connectivity strength in each interval was defined as the mean of connectivity strengths within a specific distance interval.

### Pattern-Classification Analysis Based on MVPA

A multi-voxel pattern analysis (MVPA) procedure was applied to classify conscious state in all the subjects. For each brain region of the whole-brain network, three nodal topological metrics were obtained: connectivity strength, betweenness, and degree. For each subject, a feature vector was constructed for pattern classification by concatenating the three nodal topological metrics of all brain regions within each of the ten functional networks. All subjects were divided into non-overlapped training and testing samples. Using the feature vectors, we applied a linear support vector machine (SVM) approach to perform classifier training and generalization testing^13^. For the classifier training, feature vectors of the training samples were used to train three linear SVM classifiers based on the one-versus-rest method (e.g., VS/UWS vs. MCS and controls)^25^. The purpose of this procedure was to deal with the multi-class classification problem. For generalization testing, these three SVM classifiers were applied to the feature vectors extracted from test samples. Hence, three SVM output scores were obtained. Using a previously described loss-based decoding method^25^, we assigned the test subject a class label (i.e., VS/UWS, or MCS, or control) corresponding to the maximal score.

The leave-one-out-cross-validation (LOOCV) strategy was used to evaluate performance of the three SVM classifiers. In the calculations, each subject was used as the test sample in turns while the remaining subjects were used as training samples to train the three SVM classifiers. Classification performance of the classifiers was quantified using the accuracy obtained from the cross-validation estimations. Statistical significance of classification accuracy was assessed by conducting a nonparametric permutation test^13^,^26^. In each permutation procedure, classification accuracy was computed by performing the LOOCV strategy described above while randomly assigning label orders to the training and test samples. Corresponding accuracies were obtained after 1,000 permutations and used to build a null distribution. The *p*-value represented the proportion of the accuracies in the null distribution that were greater than or equal to the actual classification accuracy, which was obtained using the non-permutated training samples.

Classification performance was most optimal when features in the FPN and DMN were combined (see Results). Therefore, the same MVPA steps were performed with these features for classification between groups (i.e., control vs. MCS, control vs. VS/UWS, or MCS vs. VS/UWS) based on the LOOCV strategy. The level of significance for classification accuracy was set at *p* < 0.05 based on the nonparametric permutation test as above. The sensitivity and specificity of the classification were also calculated. The sensitivity represents the proportion of MCS/VS/VS correctly classified, while the specificity represented the proportion of control/control/MCS correctly classified between groups of MCS and control/VS and control/VS and MCS.

### Nodal Characteristics

The contribution of each node to the prediction of conscious states was dependent on its absolute weight in the feature vectors^13^. For each node, its weight was computed by summing the feature weights (i.e., SVM weights for the features) of its corresponding three features/topological metrics in the three SVM classifiers. We took its absolute value to represent the relative contribution to the prediction of conscious state.

### Statistics Analysis

Two-way analysis of variance (ANOVA) was used to examine the effect of conscious state (VS/UWS, MCS, and control) and brain network on the topological metrics of network connectivity strength, betweenness, and degree. One-way ANOVA was performed to determine the effect of conscious state on nodal connectivity strength within the FPN and DMN at different spatial distances. Tukey post-hoc tests were used to carry out significant comparisons. Pearson’s correlation analysis was used to assess the relationship of topological metrics between the FPN and DMN or between different brain regions within these two networks in the DOC patients while using the average topological metrics in controls as the baseline (Bonferroni corrections were used for multiple comparisons).

A nonparametric permutation test was performed to determine which brain regions exhibited altered nodal topological metrics within the FPN or DMN^13^,^26^. Differences between groups (VS/UWS, MCS and control) for each nodal topological metric within the FPN and DMN was represented as the *F*-value from one-way ANOVA. The average *F*-value across the three topological metrics for each node (FPN = 25 nodes, DMN = 58 nodes) was used to perform nonparametric permutation tests. Initially, all subjects in the three groups were combined together and randomly re-sampled into three new groups with the same number of subjects in each group. A new average *F*-value for each nodal topological metric within the FPN and DMN was then obtained after performing the ANOVA among the 3 re-sampled groups. This process was repeated 10,000 times resulting in 10,000 average *F*-values corresponding to each node within the FPN and DMN. These values were used to build a null distribution. The *p*-value of the nodal average *F*-value was calculated as a percentile in the null distribution. Multiple comparisons were corrected using FDR correction (*p*-value < 0.05). The *p*-values of the correlation analysis are presented as the corrected value after Bonferroni corrections. Group data are presented as the mean ± standard deviation in the text.

## Results

### Network Level Topological Metrics

**Figure 2:**
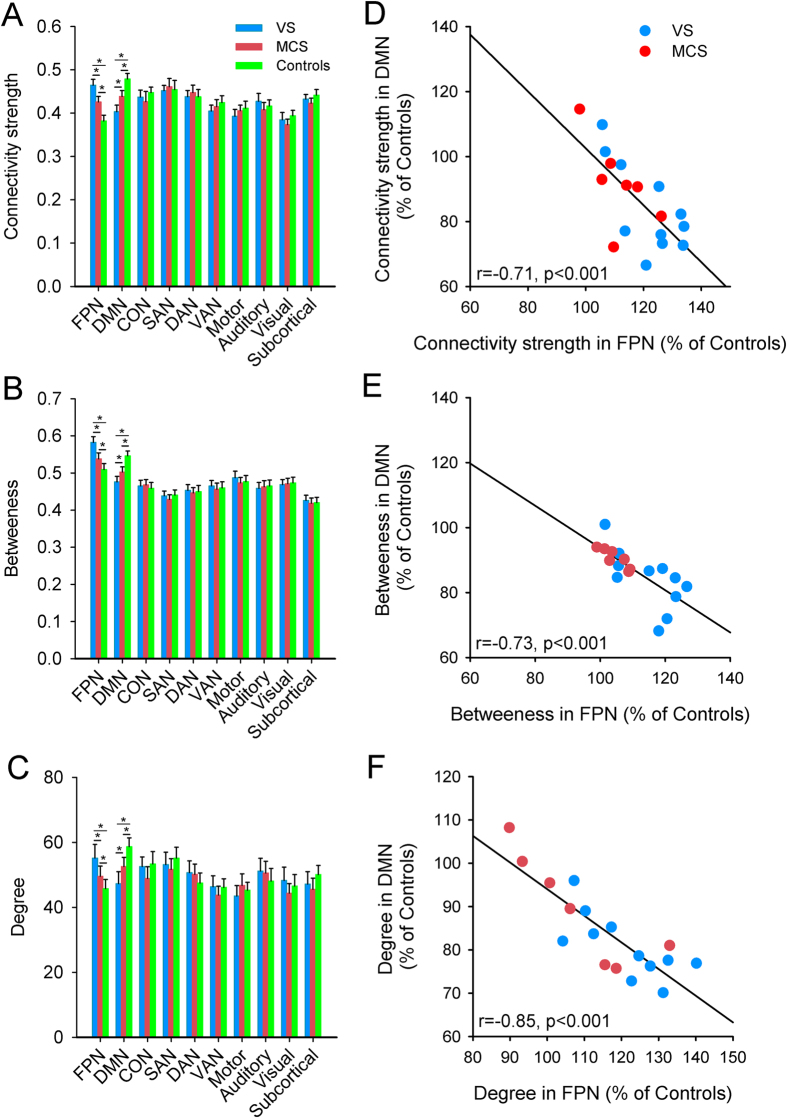
Topological metrics of each functional network by group: (**A**) connectivity strength, (**B**) betweenness, and (**C**) degree. The relationship between the network topological measures in the FPN and DMN in DOC patients with the controls as baseline: (**D**) connectivity strength, (**E**) betweenness, and (**F**) degree. Error bars indicate standard error. *p < 0.01.

The two-way ANOVA showed a significant main effect for conscious state (*F*_2,260_ = 52.21, *p* < 0.001), but not for brain network (*F*_9,260_ = 1.51, *p* = 0.14), on network connectivity strength (Fig. 2A). There was also an interaction between conscious state and brain network (*F*_18,260_ = 3.54, *p* < 0.001). Post-hoc tests indicated increased network connectivity strength within the FPN (*p* < 0.01) and decreased strength within the DMN (*p* < 0.01) in both the VS/UWS and MCS groups compared to controls. In addition, network connectivity strength for the VS/UWS group was significantly increased within the FPN (*p* < 0.01) and decreased within the DMN (*p* < 0.01) compared to the MCS group. Network connectivity strength was also increased within the FPN but decreased within DMN for the VS/UWS and MCS groups compared to the control group. Similar trends were evident in the other two topological metrics, betweenness (*p* < 0.001; Fig. 2B) and degree (*p* < 0.001; Fig. 2C), supporting the hypothesis that topological metrics were changed within the FPN and DMN in DOC patients. We also found significant negative correlations between the FPN and DMN in the network connectivity strength (*r* = −0.71, *p* < 0.001; Fig. 2D), betweenness (*r* = −0.73, *p* < 0.001; Fig. 2E), and degree (*r* = −0.85, *p* < 0.001; Fig. 2F) in the DOC patients. These results indicated that topological metrics of FPN and DMN were anti-correlated across states of consciousness.

### FPN and DMN Regions with Anti-Correlated Topological Properties

To determine if anti-correlated properties are also observed in specific regions within the FPN and DMN, we initially identified brain regions exhibiting significantly altered topological metrics within the FPN and DMN in DOC patients. These regions are located in the bilateral dorsolateral prefrontal cortices (DLPFC, BA 9 and 46) of the FPN (*p* < 0.05) and bilateral precuneus (BA 7) of the DMN (*p* < 0.05). The nodal topological metrics between the DLPFC and the precuneus regions were negatively correlated after Bonferroni corrections: strength (*r* = −0.67, *p* < 0.001), betweenness (*r* = −0.62, *p* < 0.001), and degree (*r* = −0.63, *p* < 0.001).

### Distance-Related Connectivity Strength

**Figure 3:**
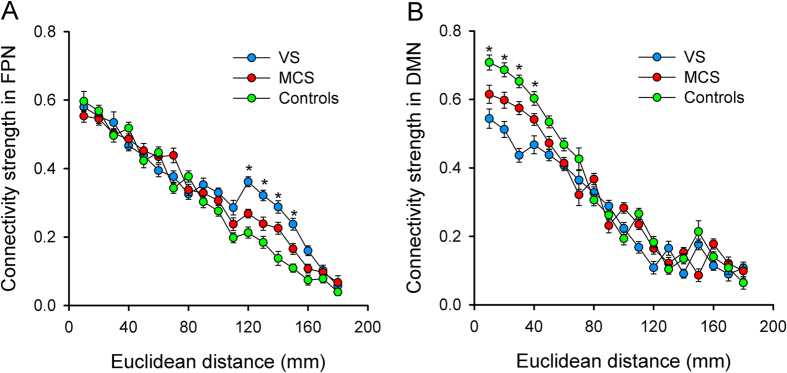
Nodal connectivity strength across all functional connections as a function of the Euclidian distance between each node and one of the other nodes within the whole-brain network by group: (**A**) FPN and (**B**) DMN. Error bars indicates standard error. *p < 0.01.

Results of the one-way ANOVA showed that network interactions related to connectivity strength are spatially distance dependent. This revealed that nodal connectivity strength in long-distance connections (120–150 mm) within the FPN was different between groups (*p* < 0.01; Fig. 3A). Post hoc tests indicated that nodal connectivity strength was stronger in these long-distance connections in both the VS/UWS and MCS groups relative to the control group (*p* < 0.01) and stronger for the VS/UWS group relative to the MCS group (*p* < 0.01). Nodal connectivity strength in short-distance connections (10–40 mm) within the DMN was also different between groups (*p* < 0.01; Fig. 3B). In contrast to the FPN, however, post-hoc tests showed that nodal connectivity strength in both the VS/UWS and MCS groups was weaker in these short-distance connections compared to the control group (*p* < 0.01) and weaker for the VS/UWS group compared to the MCS group (*p* < 0.01). Taken together, these results demonstrate inverse trends for distance and connectivity strength between FPN and DMN networks, suggesting different patterns of signal integration in individuals with impaired consciousness. That is, distant FPN connections exhibit stronger connectivity and closer DMN connections show less connectivity in VS/UWS and MCS patients compared to controls or in MCS compared to VS/UWS patients.

### MVPA Pattern-Classification

To determine if connectivity in specific network(s) discriminate conscious state, we applied a multivariate pattern analysis (MVPA) classification algorithm to each of the 10 functional networks. The specific objective was to determine if functional connectivity patterns within the FPN and DMN, and not the other eight networks, could be used to discriminate conscious state. Results indicated classification accuracy was significantly above chance level (33.3%) not only for the FPN (accuracy 65.5%, permutation test *p* < 0.001; 9/11, 4/7, 6/11 correct prediction for controls, MCS, and VS/UWS separately) but also for the DMN (accuracy 72.4%, permutation test *p* < 0.001; 10/11, 4/7, 7/11 correct prediction for controls, MCS, and VS/UWS separately). Classification accuracy was not above chance level (33.3%) for any of the other eight networks (unadjusted *p* > 0.05; Fig. 5A).

**Figure 4:**
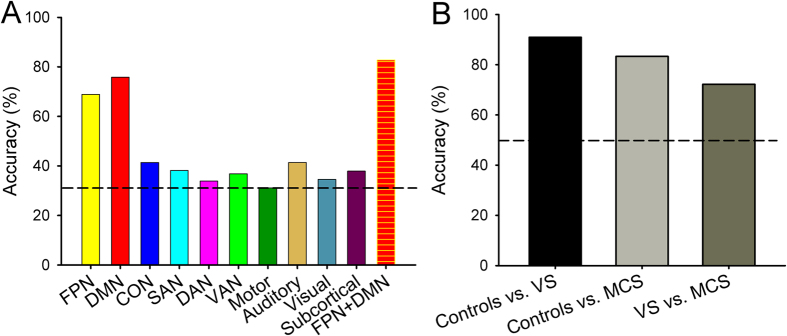
Mean classifier accuracy. (**A**) mean accuracies for classifying individual subject into groups (VS/UWS, MCS and controls) were obtained based on the connectivity patterns within each functional network and then combining connectivity patterns within the FPN and DMN. (**B**) mean accuracies for classification between groups were obtained based on the combining connectivity patterns within the FPN and DMN. The dashed lines represent the chance level (33.3% in **A** and 50% in **B**).

**Figure 5:**
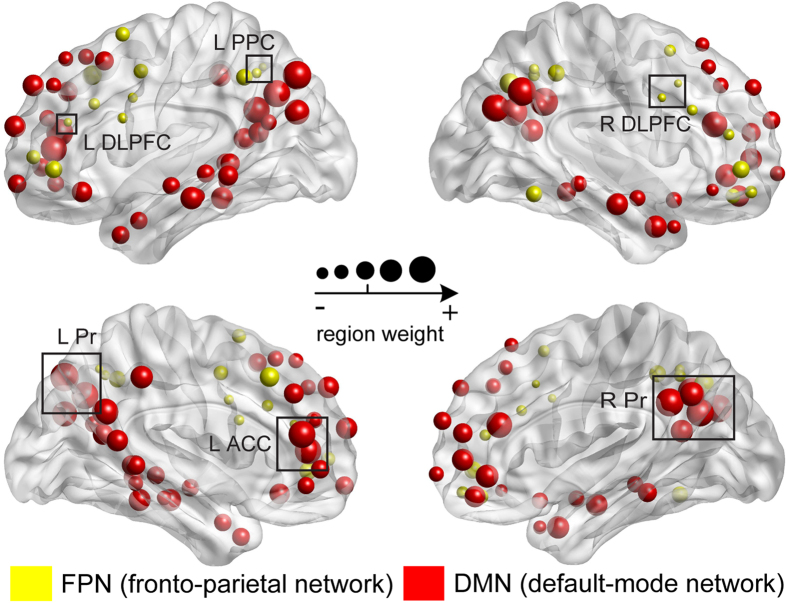
Mean SVM weight maps for the regions within the FPN (yellow) and DMN (red). The radius of a node is scaled with its region weight by summing its corresponding feature weight of the three topological metrics within the three SVM classifiers. Notably, the absolute value of the nodal weight determines its contribution to the classification performance. Abbreviations: PPC, posterior parietal region; DLPFC, dorsolateral prefrontal cortex; Pr, precuneus; and ACC, anterior cingulate cortex; L(R), left (right) hemisphere.

Connectivity patterns within the FPN and DMN networks were combined to further discriminate conscious state in DOC patients. The repeated classification analysis showed that accuracy was increased to 82.7% (permutation test *p* < 0.001; 10/11, 6/7, 8/11 correct prediction for controls, MCS, and VS/UWS separately; Fig. 4A), which was higher than that derived from the connectivity patterns within either the FPN or DMN, suggesting that connectivity between these networks is more effective in classifying conscious state than either network alone. In addition, classification analysis based on functional connectivity patterns within the FPN and DMN also exhibited significantly higher performance than chance level (50%) in distinguishing VS/UWS patients from controls (accuracy = 90.9%, permutation test *p* < 0.05; sensitivity = 81.8%; specificity = 100%; Fig. 5B), MCS patients from controls (accuracy = 83.3%, permutation test *p* < 0.05; sensitivity = 71.4%; specificity = 90.9%; Fig. 5B), or VS/UWS patients from MCS patients (accuracy = 72.2%, permutation test *p* < 0.05; sensitivity = 72.7%; specificity = 71.4%; Fig. 4B).

The contribution of different brain regions to the prediction of conscious state was determined by plotting nodal weights (radius of a node is proportional to the corresponding region weight) for all brain regions within FPN and DMN (Fig. 5). Brain regions within the FPN exhibited mainly negative weights, while regions within the DMN exhibited mostly positive weights. The largest contribution to conscious state prediction was from the bilateral dorsolateral prefrontal cortex (DLPFC, BA 9 and 46) and left posterior parietal region (PPC; BA 5) within the FPN, and the bilateral precuneus (Pr; BA 7 and 31) and left anterior cingulate cortex (ACC; BA 32) within the DMN (Fig. 5). Taken together, these results indicate that connectivity patterns between the FPN and DMN predict conscious state.

## Discussion

Findings from the present study reveal abnormal FPN and DMN topological properties in DOC that are anti-correlated between networks. This anti-correlation was observed between nodal topological metrics in the dorsolateral prefrontal cortex of the FPN and precuneus of the DMN. In addition, we found architectural differences between networks in subjects with DOC. Specifically, altered nodal connectivity within the FPN mainly occurred in long-distance connections, while it primarily occurred in short-distance connections within the DMN. Classification analysis indicated that FPN and DMN connectivity patterns discriminate conscious state. These results confirm previous findings implicating the involvement of FPN and DMN in DOC and highlight specific interactions in network-level topology that may play a role in mediating consciousness.

In this study, DOC patients exhibited abnormal FPN and DMN functional connectivity, a finding that is consistent with results of previous inter-nodal correlation analyses in MCS^4^,^27^, VS/UWS^3^ and coma patients^4^. Findings of the current study demonstrate that these networks show a distinct modulation of topological properties in DOC. Additional support for the involvement of these networks comes from analyses used to predict conscious state. VS/UWS, MCS, and control groups could be discriminated on the basis of FPN and DMN connectivity patterns and not connectivity patterns in the other eight networks. This is in line with previous R-fMRI studies demonstrating a disruption in topological parameters of brain regions within the DMN and FPN in impaired consciousness^6^,^7^,^8^. A recent EEG study also reported altered topological properties within the FPN following propofol-induced unconsciousness^28^.

Some studies have not detected differences in global level topological properties between comatose patients and healthy controls^5^ or between MCS and VS/UWS patients^6^. It is possible that these properties may be not sufficiently reflected at the global network level because some functional networks show decreased properties, while others show simultaneously increased properties^5^. This interpretation is supported by the anti-correlated nature of network-level topological properties within the FPN and DMN across different conscious states in the current study, in which the increase of FPN topological properties may perform the counterpart of the decrease of DMN topological properties. The implication here is that topological properties at the network level, rather than across the global/whole-brain network, may contribute in some way to the determination of clinical status in individuals with DOC.

The FPN and DMN exhibit antiphasic activation patterns^2^,^29^,^30^,^31^. A postulate forwarded by previous work is that this pattern of activation may account, in part, for the contribution of the FPN and DMN to impaired consciousness during hypnosis^32^ and in VS/UWS^3^. Consistent with this interpretation, the topological metrics of FPN and DMN were anti-correlated across DOC patients, which may be due to the increased importance of cortical nodes switching from the DMN to FPN after impaired consciousness^5^.

A novel finding from the current study is that the disruption in nodal connectivity strength within FPN and DMN is spatially distance-dependent. FPN regions with altered connectivity strength corresponded to long-distance connections, while DMN regions with altered connectivity strength related to short-distance connections. These findings are consistent with previous work that shows connectivity strength is decreased in short-distance connections but increased in long-distance connections for subjects in an anesthetic-induced unconscious state^22^. Similar to the inverse relationships between topological properties of these two networks with conscious impairment, the functional architecture of each network is also diametrically organized.

Distinct anti-correlated brain regions within either network were also evident in this study. Classification analysis showed that dorsolateral prefrontal cortex within the FPN and precuneus within the DMN made the largest contribution to predicting conscious state. The dorsolateral prefrontal cortex, a region within the FPN, has been suggested to play a key role in conscious processes^33^,^34^,^35^, especially in the presence of conscious demands^36^ and during visual perception^37^. A previous R-fMRI study also demonstrated that coma patients exhibited increased nodal topological properties in the frontal cortex (BA 9 and 46)^5^. The observed reduction in topological properties of precuneus is also consistent with previous work implicating this region in conscious impairment^4^. In addition, Achard *et al*. reported a significant reduction in the precuneus of coma patients^5^, and Crone *et al*. found decreased local efficiency of the precuneus in impaired consciousness^6^.

The present study has some noted limitations. First, segmentation of the brain image of DOC patients is complicated by distortions in different types of tissues (e.g. white matter, CSF) during automated segmentation^38^. To overcome this challenge, DOC patients with larger hematoncus and malacostic foci, moderate to severe hydrocephalus, or severe brain atrophy were excluded. Although functional connectivity is related to underlying brain anatomy^39^ and the radiological analysis with diffusion-weighted imaging would be helpful to explain the differences found in the rs-fMRI data reported here, the diffusion images are more easily distorted due to the susceptibility and brain deformation compared to rs-fMRI data. Further multimodal studies should combine rs-fMRI data with structural MRI (e.g. diffusion tensor imaging data) to explain the results in this study. Second, like most imaging studies on clinical populations, there is a great deal of heterogeneity with respect to symptoms, duration of illness, and use of medications among patients. These factors may bias topological properties of functional brain networks^40^,^41^. Although attempts were made to minimize these effects by selecting DOC patients with only hypoxic ischemic encephalopathy in an non-sedated state, a larger sample of DOC patients is needed in future research to replicate the findings reported here. Third, an imperfect set of putative functional regions was selected in this study. The brain template used contains 264 regions that were identified from a fMRI meta-analysis^10^. Other methods for constructing high resolution functional brain networks involve selecting nodes on the basis of image voxel or cortical markers (e.g., gyri and sulci). Brain regions defined using this method, however, do not align well with functional regions potentially biasing the findings of network analysis and functional connectivity MVPA^42^,^43^. Fourth, previous studies have shown that topological properties are different between individuals in MCS and VS/UWS^5^,^6^, making it possible to identify a pattern for discriminating between states^44^. However, misdiagnosis rates in VS/UWS and MCS patients based on behavioral observation are relatively high^45^, making classification with machine learning approaches particularly challenging. Although the CRS-R was administered on multiple occasions to verify conscious state, future work may incorporate more robust tools for evaluating conscious state. Fifth, since excessive head movement would affect image realignment and segmentation^8^ and, in turn, compromise accurate normalization on the brains of DOC patients, it is important to control the selection of patients carefully to prevent the bias during data analysis. Similar to previous studies^8^, 6 patients (2 VS/UWS and 4 MCS) had to be excluded due to excessive head movement. Although data from 18 patients were included in analyses, it could be argued that our sample is relatively small. Thus, future work can expand on these findings while including a larger sample to reaffirm the results of this study. Finally, 15–20 minutes of scanning is suggested for rs-fMRI connectivity analysis, which requires that subjects remain awake and motionless over the entire duration. DOC patients have difficulty remaining awake and maintaining a stable head position for extended periods of time because of their cognitive and physical impairments^4^. To obtain the highest quality data across the sample of subjects given these challenges, functional imaging was restricted to 8 minutes (240 volumes). The scanning time used in this study is comparable to previous studies involving this clinical population (e.g. 250 scans in Demertzi *et al*.^44^ and 200 scans in Vanhaudenhuyse *et al*.^4^). Furthermore, Van Dijk *et al*. conducted a series of experiments to examine the influence of different acquisition parameters on rs-fMRI, such as run length (2–12 min), temporal resolution (2.5 or 5s), spatial resolution (2 or 3 mm), and subject state (fixation, eyes closed rest, and eyes open rest)^46^. They found that the 4-min run lengths were sufficient to estimate connectivity reliably and also that position within the run had minimal effect on the estimate.

## Conclusion

In summary, this study provides evidence of network-level abnormalities contributing to DOC. Our results demonstrate that there are inverse trends in the relationships between conscious impairment and the respective topological properties of the FPN and DMN. These relationships were particularly evident in nodal topological metrics of the dorsolateral prefrontal cortex of the FPN and precuneus of the DMN. Altered connectivity strength in DOC was distance dependent, with most disruptions appearing in long-distance connections within the FPN but occurring in short-distance connections within the DMN. The combination of FPN and DMN network-level topology patterns strongly predicted conscious state. Collectively, these findings imply distinct interactions between FPN and DMN that may play a role in mediating consciousness and, therefore, provide a potential biomarker of conscious state.

## Additional Information

**How to cite this article**: Long, J. *et al*. Distinct Interactions between Fronto-Parietal and Default Mode Networks in Impaired Consciousness. *Sci. Rep.* **6**, 38866; doi: 10.1038/srep38866 (2016).

**Publisher's note:** Springer Nature remains neutral with regard to jurisdictional claims in published maps and institutional affiliations.

## Figures and Tables

**Table 1:** Characteristics of subjects in this study.

Characteristics	VS/UWS	MCS	Controls	*p*-value
Clinical Features				
Age (years old)	41.8 ± 19.3	42.2 ± 22.4	34.4 ± 11.1	0.53^b^
Gender (male/female)	9/2	5/2	8/3	0.28^a^
CRS-R				
Auditory function	0.54 ± 0.52	1.2 ± 0.48	—	0.008^b^
Visual function	0.09 ± 0.31	2.3 ± 0.95	—	<0.001^b^
Motor function	0.91 ± 0.83	3.7 ± 1.2	—	<0.001^b^
Verbal function	0.54 ± 0.52	1.7 ± 0.48	—	<0.001^b^
Communication	0 ± 0	0.14 ± 0.37	—	0.09^b^
Arousal	1.6 ± 0.46	2.2 ± 0.48	—	0.02^b^
Total score	4.1 ± 1.3	11.4 ± 2.5	—	<0.001^b^

^a^χ^2^ – test.

^b^Two-sample *t*-test.
